# Reproducibility and Validity of a Semi-Quantitative Food Frequency Questionnaire for Children Aged 6–12 in Western China

**DOI:** 10.3390/nu15040856

**Published:** 2023-02-08

**Authors:** Yujie Qin, Hongyang Deng, Mengnan Lu, Yue Cheng, Baibing Mi, Yanfeng Xiao, Jing Zhou

**Affiliations:** 1 Department of Pediatrics, The Second Affiliated Hospital of Xi’an Jiaotong University, Xi’an 710004, China; 2 Department of Pediatrics, Second School of Clinical Medicine, Health Science Center, Xi’an Jiaotong University, Xi’an 710004, China; 3 Department of Epidemiology and Biostatistics, School of Public Health, Health Science Center, Xi’an Jiaotong University, Xi’an 710061, China

**Keywords:** food frequency questionnaire, reproducibility, validity, diet surveys, child, western China

## Abstract

The Food Frequency Questionnaire (FFQ) is often used to assess dietary intake in large-scale epidemiological studies. This study aimed to evaluate the reproducibility and validity of the FFQ newly developed for children aged 6 to 12 in western China. A total of 133 children were included in the analysis, and all the children and their caregivers completed the FFQs twice with a three-month interval period, and three 24 h recalls were carried out one month after the first FFQ. We assessed the relative validity and reproducibility using various methods, such as the Spearman correlation coefficient, intra-class correlation coefficient, weighed Kappa, quartile agreement, and Bland–Altman analysis. The Spearman correlation coefficients for food ranged from 0.30 to 0.84, and for nutrients from 0.46 to 0.82 regarding reproducibility. The food intra-class correlation coefficients ranged from 0.20 to 0.85, while nutrients’ ranged from 0.37 to 0.75. In terms of relative validity, the average Spearman correlation coefficients for food were 0.20, and 0.30 for energy and nutrients. The energy-adjusted and de-attenuation coefficients were calculated. Moreover, the average percentage of participants misclassified into the extreme quartile for food and nutrients was 8.0% and 7.0%, respectively. Weighted Kappa values indicated acceptable agreement between the FFQs and 24 h recalls. Furthermore, the percentage of results in the limits of agreement (LOA) were all above 93.0%. In conclusion, The FFQ showed good reproducibility and acceptable relative validity for assessing the dietary intake of children aged 6–12 in western China.

## 1. Introduction

Childhood is a critical stage of growth and development and is important for forming eating habits. A previous study reported that nutritious diets in early and mid-childhood were associated with higher height and fat-free mass index [1]. Balanced nutrition has positive effects on childhood growth and body composition. Additionally, childhood nutrition plays a significant role in sexual maturation [2] and obesity, which leads to severe complications such as fatty liver, hyperuricemia, and insulin resistance. It increases the risk of coronary heart disease, diabetes, and premature death [3]. Therefore, it is essential to evaluate children’s diet and nutrition intake using various methods, including a food frequency questionnaire (FFQ), 24 h dietary recalls, and weighed food records.

The 24 h dietary recall and weighed food record provided relatively accurate assessments of short-term dietary intake [4]. Due to time and labor requirements, weighed food records are rarely used in large-scale dietary surveys, even though they provide a more accurate assessment of the respondents’ food intake [5]. The FFQ covers a longer period of dietary history and is frequently used to estimate long-term dietary exposures, mainly because of the applicability of large-sample epidemiological studies [6,7,8]. Due to the different ethnic, social, and cultural backgrounds of the study population, the type of food consumed varies significantly. Therefore, each FFQ must be designed for the geographic region and dietary preferences of the target population [9]. Children present a greater challenge for dietary surveys than adults because of their greater nutritional needs and a greater variety of food sources. Some FFQs have been validated for use with children and adolescents in countries including New Zealand [10], Peru [11], Mexico [12], and Italy [13]. In China, the general adult population and some special populations such as lactating mothers [14] and pregnant women [15] use mature FFQs. However, FFQs for children are relatively scarce. Liu [16] and Ma [17] developed and validated FFQs for children aged 12–17 and preschoolers, respectively; the FFQs they designed both exhibited good reproducibility and acceptable relative validity. There are still few validated FFQs for use with Chinese primary school-aged children. It is an urgent necessity that the FFQ for this age group is available for nutrition assessment of children in China. A validated FFQ for this age group can have a positive impact on children’s nutrition assessment and provide a good tool for epidemiological studies on diet and its relationship with the onset of diet-related diseases in Chinese children.

This study aimed to develop and validate a semi-quantitative FFQ for children aged 6 to 12 years in western China. We assessed reproducibility and validity using two FFQs and three 24 h recalls, a more precise method for evaluating short-term dietary intake.

## 2. Materials and Methods

### 2.1. Study Population

This semi-quantitative FFQ validation and reproducibility study was undertaken as part of a cohort study on the growth and diet of school-age children. Using the cluster sampling method, we conducted this study on children aged 6–12 years in Xi’an, Shaanxi Province, a representative city in western China, between June 2021 and September 2022. The children were from four primary schools with different teaching levels, two in urban and two in rural areas. Six classes, three from the first to the third and three from the fourth to the sixth grades, were randomly selected. We invited 184 children to participate in the study, 95 boys and 89 girls; however, 51 students were excluded due to lack of parental consent, incomplete dietary records, or FFQs. Eventually, we enrolled 133 children in the study. The inclusion criteria were healthy children who had lived in the local area for more than 12 months and whose diets had not changed significantly over the past year. The Xi’an Jiaotong University ethics committee approved this study, and all guardians provided a signed electronic informed consent form before recruitment.

### 2.2. Study Design

**Figure 1:**
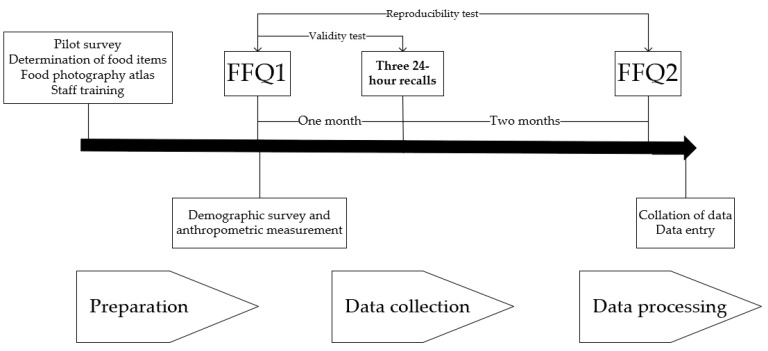
The design of the reproducibility and relative validity study among 133 children in western China.

Figure 1 shows the study design and schedule. Before collecting data from participants, we surveyed the dietary habits of people in western China to identify food items, created a food photography atlas, and trained interviewers and class teachers. Throughout the study, we collected two FFQs and three 24 h recalls from the participants and their caregivers. In the reproducibility study, the first FFQ (FFQ1) was administered at enrollment, along with demographic questionnaires, anthropometrics, and measurements. The second FFQ (FFQ2) was conducted 3 months later. Three 24 h recalls were collected 1 month after FFQ1, including 2 weekdays and 1 weekend to validate the FFQ. FFQs and three 24 h recalls were administered through interviews with specialized trained project staff. They were required to record the portion sizes of ingredients in the dishes using standard kitchen scales. Subsequently, two staff members were assigned to collate and enter the data obtained. Qualified and experienced nutritionists and dietitians were in charge of this study’s preparation, information collection, and data entry.

### 2.3. Food Frequency Questionnaire (FFQ)

We developed a semi-quantitative FFQ of 120 food items divided into 12 food groups: cereals and potatoes, meat, eggs, aquatic products, milk and milk products, soy and soy products, vegetables, fruits, nuts, snacks, beverages, and oil (Supplementary Table S1). Dietary Guidelines for Chinese School-aged Children [18] and the eating habits of children in western China informed the development of food items. Participants and their caregivers were asked to recall their typical consumption frequency and portion size during the past year for each item. On the FFQs, there were eight frequency options provided, including “never”, “less than one time per month”, “1–3 times per month”, “1–2 times per week”, “3–4 times per week”, “5–6 times per week”, “once per day”, and “twice or more times per day”. The portion size of most foods listed on the questionnaire was specified as large, medium, or small sized. The portion size categories were created based on an analysis of the pilot 24 h recall data and Dietary Guidelines for Chinese School-aged Children. For some foods that people are used to eating whole, such as eggs and dumplings, the consumption was described by numbers. Color photographs of the food portions were used to facilitate the assessment of their sizes to improve the accuracy of the estimation. The foods in each photograph were served on uniform-sized plates or bowls, which are common in local markets. The size of each food portion was compared to a credit card that the participants were familiar with. Additionally, the weight of the food in the image was also marked. The camera settings were fixed at the same angle when capturing pictures of different portions of the same food. The questionnaire also included extra dietary information such as eating location, timing, and dietary supplements. This analysis was limited to food and nutrients derived from the diet, so these data were not analyzed.

### 2.4. Twenty-Four Hour Recall

Three 24 h recalls were selected as reference data to analyze relative validity. Participants’ caregivers and their class teachers received instructions on how to complete the 24 h recall upon enrollment. The caregivers were instructed to record and estimate the portion size of all foods and beverages consumed in one day. A photographic atlas containing food images of various portion sizes and everyday household cooking utensils was provided to aid in estimating portion size. The dietary record data contained single foods (e.g., apples, walnuts, and cakes) and mixed foods (e.g., stewed chicken with mushrooms). All mixed dishes were transformed into their original single components according to the methods of food preparation. Some children ate breakfast and lunch at school during the weekdays, prepared and distributed by the canteen staff. The class teachers and trained instructors completed this part of the dietary record together. The following morning, the interviewers confirmed to the children that nothing had been missed, including the snacks and beverages they had consumed. The interviewers conducted a final review and check of the first 24 h recall. The same procedure was followed for the subsequent two 24 h recalls.

### 2.5. Food and Nutrient Assessment

For the FFQs, the sum of daily food consumption was determined by multiplying the frequency of daily food consumption by the amount consumed each time. When the frequencies were within the range of values, the averages of the highest and lowest frequencies were taken. The nutritional value of each food item was calculated by matching the food codes in the China Food Composition Tables (6th and 2nd editions) [19,20]. For example, a participant ate beef 1–3 times per month in the past year, consuming 50 g each time. One to three times per month was converted into 2 times per month, and then transformed to 0.071 times per day. The daily consumption of beef was 3.55 g (0.071 × 50 = 3.55 g). The total daily energy and nutrient intake of the child was calculated as the sum of the energy and nutrient values of 120 food items. For three 24 h recalls, the data were inputted into the Nutrition Calculator (v2.8.0(k), Institute for Nutrition and Food Security, Chinese Center for Disease Control and Prevention), a dietary software based on the Chinese Food Composition Tables. Daily food consumption and nutrient intake were calculated using the dietary software. If one food was not listed in the tables, the nutritional value was determined using foods with similar ingredients. The nutrients of raw materials were calculated in accordance with the recipe once the item did not have nutritional equivalency with another food included in the nutrient database. Data on food groups and nutrient intake derived from the dietary software and FFQs were imported into Microsoft Excel for statistical analysis.

### 2.6. Statistical Analysis

IBM SPSS Statistics, version 25.0 (SPSS Inc., Chicago, IL, USA), and R software, version 4.0.3 (The R Foundation for Statistical Computing, Vienna, Austria), were used for the statistical analysis. *p* < 0.05 was considered to be statistically significant. All categorical variable data, such as parental education status and monthly family income level, were expressed as frequencies (*n*) and percentages (%). Continuous variable data, such as age, height, and weight, were expressed as mean and standard deviation (SD). The data on daily food consumption and nutrient intake from FFQs and 24 h recalls were non-normal and described by the median, 25th, and 75th percentiles, respectively. The Wilcoxon signed-rank test was used to compare the differences in food consumption and nutrient intake between the two FFQs and between the two methods (FFQ1 and average of three 24 h recalls)

The original and log_10_-transformed data were examined for normality; none of them conformed to the normal distribution. Spearman correlation coefficients (SCC) and intra-class correlation coefficients (ICC) were calculated to test the reproducibility between the two FFQs. Spearman correlation coefficients were used to evaluate the relative validity between FFQ1 and the 24 h recall (average of three 24 h recalls). The energy-adjusted intake of food and nutrients was calculated using Willett’s residual method to eliminate variation due to energy [21]. The Spearman correlation coefficients were de-attenuated to reduce the effect of within-person variations from 24 h recalls, using the equation R = r(1 + λ/nx)^0.5^, where λ is the ratio of within- and between-person variances, nx is the number of replicates [22], and in this study nx is 3. The correlation coefficient of ≥0.50 was considered a good outcome, between 0.20 and 0.49 was acceptable, and <0.20 was a poor outcome [23].

Quartile agreement was used to assess the reproducibility and relative validity of the FFQ. By sorting the food and nutrient intake values of the two FFQs and the 24 h recall, the proportion of children correctly classified into the same, adjacent, or extreme quartiles was computed (average of three 24 h recalls). To investigate the classification agreement further, we figured out the weighted kappa. The weighted kappa values of >0.60, 0.20 to 0.60, and <0.20 represented good, acceptable, and poor agreement, respectively [23]. Furthermore, Bland–Altman plots were performed to examine the agreement between FFQ and 24 h recall for energy and nutrient intake [24]. The level of agreement could be estimated by determining whether the majority of data points fell within the 95% limits of agreement (LOAs) and the mean line close to 0.

## 3. Results

### 3.1. The Characteristics of the Participants

Table 1 shows the characteristics of the study population. Two FFQs and three 24 h recalls were completed by 133 parent–child pairs, of which 68 were boys. The mean age of these children was 9.3 years, the mean height was 139.3 cm, the mean weight was 35.1 kg, and the mean BMI was 18.0 kg/m^2^. According to the Chinese standard for children and adolescents [25], 4.5%, 17.8%, and 15.8% of children were underweight, overweight, and obese, respectively. In addition, over half of the participants had an average family income of CNY 5001–10,000 per month. There were no significant differences in age, BMI, parental education level, or average family income between girls and boys (*p* > 0.05).

### 3.2. Reproducibility

Table 2 shows the median daily intake of the food groups from the two FFQs. Compared to FFQ2, the consumption of cereals and potatoes, milk and milk products, soy and soy products, and oil was higher, and that of aquatic products, eggs, and nuts was almost equal to that estimated by FFQ1. In contrast, the intake of meat, vegetables, fruits, snacks, and beverages estimated by FFQ1 was lower than those estimated by FFQ2. The crude Spearman correlation coefficients derived from FFQ1 and FFQ2 ranged from 0.30 (vegetables) to 0.84 (milk and milk products), with an average of 0.58. The intra-class correlation coefficients for food groups ranged from 0.20 (beverages) to 0.85 (milk and milk products), and the average of the intra-class correlation coefficients was 0.52. A high proportion of participants (>70%) was classified into the same or adjacent quartiles, ranging from 71.7% (meat) to 94.0% (milk and milk products). The weighted kappa between the two FFQs ranged from 0.27 (vegetables) to 0.80 (milk and milk products).

Table 3 shows that the median intake of energy and most nutrients measured by FFQ1 were generally higher than or equivalent to those measured by FFQ2, except for dietary fiber, cholesterol, pantothenic acid, niacin, calcium, potassium, zinc, and selenium. The crude Spearman correlation coefficients for energy and nutrients ranged from 0.46 (pantothenic acid) to 0.82 (cholesterol), with an average of 0.60. The intra-class correlation coefficients ranged from 0.37 (vitamin A) to 0.75 (selenium). The percentage of agreement (the same or adjacent quartile) between the two FFQs for energy and nutrients ranged from 78.9% (vitamin C and dietary fiber) to 94.0% (cholesterol). The percentages in the extreme quartiles were all under 5%. The weighted kappa between the two FFQs ranged from 0.46 (vitamin C) to 0.73 (cholesterol).

### 3.3. Relative Validity

Table 4 compares the median daily intake of food groups from FFQ1 with the average of three 24 h recalls. Those from FFQ1 were substantially higher, except for cereals, potatoes, and eggs. The crude Spearmen correlation coefficients ranged from 0.02 (beverages) to 0.37 (milk and milk products). After adjustment for energy intake, most correlation coefficients for food groups decreased or remained unchanged, ranging from 0.02 (beverages) to 0.37 (milk and milk products). The percentage of agreement (the same or adjacent quartile) between the FFQ1 and 24 h recalls for food intake ranged from 64.7% (snacks) to 77.4% (vegetables, milk and milk products, soy and soy products). Weighted kappa ranged from 0.05 (nuts) to 0.37 (milk and milk products). An acceptable agreement was found for half of the categories.

**Figure 2:**
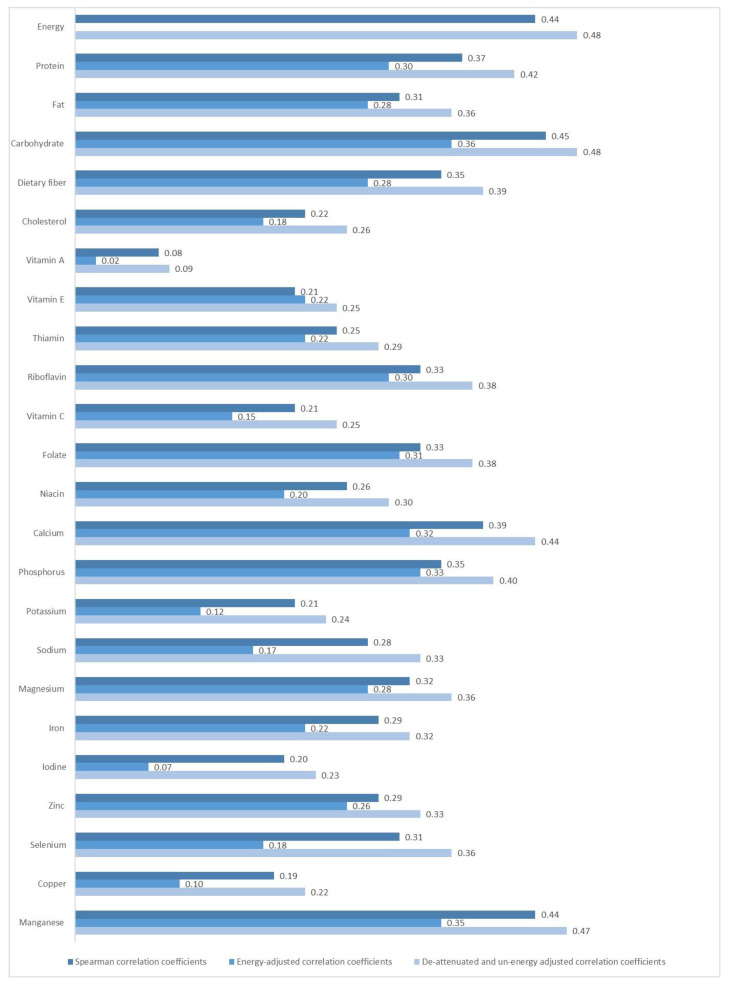
Correlation coefficients for energy and nutrients between FFQ1 and the average of three 24 h recalls in 133 children.

In summary, the median daily intake of energy and nutrients from FFQ1, except for thiamin, tended to be higher than those from the average of the three 24 h recalls (Table 5). Figure 2 and Table 5 display the correlation coefficients for energy and each nutrient. The crude Spearman correlation coefficients were highest for carbohydrate (0.45) and lowest for vitamin A (0.08). Energy adjustment led a decrease in correlation coefficients for most nutrients except for Vitamin E, and the energy-adjusted correlation coefficients ranged from 0.02 (vitamin A) to 0.38 (carbohydrate). The de-attenuated correlation coefficients for all the nutrients increased slightly and ranged from 0.09 (vitamin A) to 0.48 (energy and carbohydrate). Table 6 presents the percentage of children categorized into quartiles by energy and nutrient intake obtained from the FFQ1 and 24 h recalls. The percentage of participants divided into the same or adjacent quartile ranged from 63.2% (vitamin A) to 78.9% (manganese); the misclassification for all nutrients as extreme quartiles was rare (<10%), except for iodine (10.5%) and copper (10.5%). When evaluated using weighted kappa, most nutrients and energy had an acceptable agreement, ranging from 0.21 to 0.41. Vitamin A, vitamin E, vitamin C, potassium, iodine, and copper showed low weighted kappa values (0.08, 0.15, 0.18, 0.16, 0.11, and 0.10, respectively).

### 3.4. Bland–Altman Analysis

**Figure 3:**
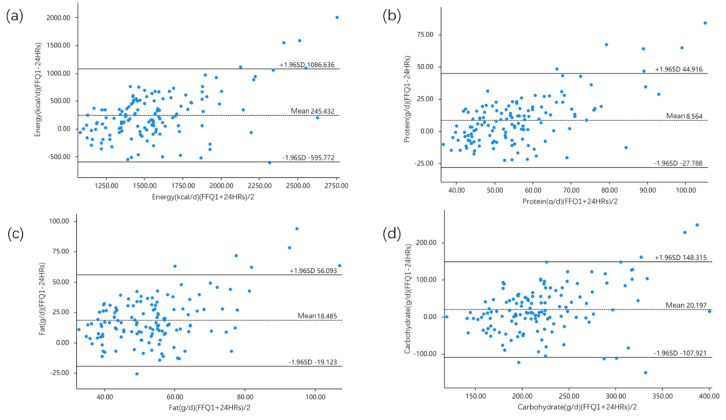
Bland–Altman plots showing the agreement in the daily intake of (**a**) energy, (**b**) protein, (**c**) fat and (**d**) carbohydrate between FFQ1 and three 24 h recalls (24 HRs) in 133 children.

The results of the Bland–Altman plots are shown in Figure 3, including energy, protein, fat, and carbohydrate. This method describes the level of discrepancy between energy and nutrient intake. According to Figure 3, most points were close to the mean line and fell within the 95% limits of agreement (LOAs). The percentage of subjects within the limits of agreement varied from 93.2% for vitamin A to 98.5% for iodine (Table 6).

### 3.5. Sensitivity Analysis

Supplementary Table S2 compares the reproducibility and validity in boys and girls using Spearman correlation coefficients, agreement by quartile, and weighted kappa. Between FFQ1 and FFQ2, the average crude Spearman correlation coefficients for energy and nutrients were slightly higher in boys (0.64) than in girls (0.56). The agreement for classifying energy and nutrient intake into the same or adjacent quartiles ranged from 76.5% to 97.1% for boys and from 73.8% to 92.3% for girls. The weighted kappa values ranged from 0.40 (vitamin A) to 0.78 (cholesterol) for boys and from 0.40 (vitamin C) to 0.77 (cholesterol) for girls.

Regarding validity, the results indicated that energy and nutrient intake assessed by the two methods were more strongly correlated in boys (average r for boys = 0.30, average r for girls = 0.28). These findings are consistent with those of reproducibility. The percentage of agreement ranged from 61.8% to 82.4% for boys and ranged from 61.5% to 86.2% for girls. An acceptable agreement according to the weighted kappa was shown for energy and most nutrients in both boys and girls.

## 4. Discussion

This study examined the reproducibility and validity of the FFQ, which was developed to evaluate the dietary intake of food groups and nutrients among children aged 6–12 years in western China. We determined the performance of this FFQ by comparing the food groups and nutrients from two FFQs separated by a three-month interval with those obtained from the 24 h dietary recalls. Overall, the results indicated that the tool was reproducible, performed satisfactorily, and its validity was acceptable.

Generally, the number of food items included in the FFQ varies between studies. According to Cade’s research, the number of food items in the FFQ was appropriate between five and 350 [6]. Using a more detailed questionnaire could dimmish the marginal gain of the information [25]. There would be little benefit to increasing the number of food items unnecessarily. The study’s FFQ included 120 food items that were deemed suitable. When assessing reproducibility and validity, Cade’s review indicated that the sample size ranged from six to 3750 [6]. In our study, 133 participants were eventually included in the analysis, which was greater than the median of 110. Furthermore, the time interval between the FFQ1 and FFQ2 varied from several months to several years, with most studies opting for a one-month to one-year interval [6]. The participants may remember their previous responses when the time interval is short, resulting in higher correlations. Three-month intervals separated the FFQ1 and FFQ to reduce bias in our study.

The average Spearman correlation coefficients for the reproducibility study were 0.58 for food, and 0.60 for energy and nutrients; similarly, the average intra-class correlation coefficients were 0.52 for food, and 0.58 for energy and nutrients, respectively. Our study’s results were comparable to those of previous studies on children and adolescents (the coefficients ranged from 0.21 to 0.69) [10,16,17,26]. However, a study of children in Puerto Rico [27] reported that the correlation coefficients for elementary school students were generally lower than those for middle school students. This may be because the older children were more knowledgeable about the names and portion sizes of the foods. Furthermore, the opposite quartiles for food, energy, and nutrients had average percentages of 3.3% and 2.1%, respectively. According to the finding of Masson [28], it is considered a successful outcome when less than 10% of the participants are classified into the highest quartile. All foods and nutrients exhibited acceptable agreement (0.27–0.75) for weighted kappa values. These findings indicate that the reproducibility of the FFQ was relatively satisfactory.

Regarding validity, the average crude correlation coefficients for food, energy, and nutrients were 0.20 and 0.30, respectively, indicating acceptable agreement. After adjusting nutrient intake for energy, validity correlations between the FFQ and the 24 h recalls decreased slightly, possibly due to individual differences in energy intake. According to a systematic review of dietary assessment methods, most correlation coefficients for children and adolescents ranged from 0.13 to 0.65 [29], similar to our results. Additionally, the validity correlation between the FFQ and 24 h recalls was lower than the reproducibility correlation coefficient. Children may forget some foods eaten outside the meal, such as beverages and snacks, if their frequency of consumption was low and inconsistent.

In this study, 72.0% of the children were classified into the same or adjacent quartiles for food and nutrients, while 8.0% for food and 7.0% for nutrients were misclassified into extreme quartiles. Eggs, snacks, copper, and iodine were the only food groups and nutrients for which 10% or more children were incorrectly classified. The level of agreement determined by quartile classification was similar or comparable to that reported in other validation studies with children and adolescents [11,16]. Six nutrients (vitamin A, vitamin E, vitamin C, potassium, iodine, and copper) exhibited poor consistency compared to most of the weighted kappa values for energy and nutrients. In this study, the weighted kappa values for validity were similar to those of a short dietary questionnaire administered to Vietnamese children [30], but slightly lower than those in other pediatric studies [31,32]. Moreover, graphically, Bland–Altman plots were drawn to examine the agreement between the two methods. The point distributions of energy and a few nutrients within the LoA for validation are shown in Figure 3, and Table 6 shows the results in percentages. Twelve nutrients and energy had over 95.0% of participant intake within the limits of agreement, and all values were above 93.0%. Although the FFQ overestimated some nutrient intakes relative to the 24 h recalls, a phenomenon observed in other studies [31,33], this could lead to the conclusion that the two methods were similar.

Our study had several strengths. Various statistical methods were used to evaluate the reproducibility and validity of the FFQ, resulting in a comprehensive evaluation of the tool. We compared the reproducibility and validity of the FFQ between boys and girls. The findings indicated that the level of agreement in boys was slightly higher than in girls, and both were generally consistent with the results for all children. In addition, as people living in western China enjoy a variety of noodle dishes, the FFQ we designed contained some local specialties familiar to people in western China. Additionally, we used the same nutrient database to calculate food and nutrient intake from the 24 h recalls and FFQs.

Some limitations of the present study should also be remarked. Because the FFQ and 24 h recall rely on self-reported data, recall bias was unavoidable [4,6]. According to the study among Peruvian children [11], there was a difference in reproducibility and validity when caregivers responded to the FFQ alone and when children and caregivers completed the FFQ together. We did not clearly distinguish between those who completed the questionnaire in this study. In addition, the FFQ covers the past 12 months, which may cause an overestimation or underestimation of the intake frequency of some seasonal foods [34], especially fruits and vegetables. Due to the irregular consumption of some foods in FFQ, they were not recorded during the 24 h recall period. These factors may alter the validity of our study. Furthermore, the Chinese frequently use a variety of seasonings in their cooking, such as salt, vinegar, and soy sauce. However, these data were not included in the FFQ. Finally, this study evaluated the relative validity of the FFQ only through the use of dietary recall, and not through the use of biomarkers [35]. Thus, the level of convincing results could be improved.

## 5. Conclusions

Growth and disease prevention in school-aged children are closely related to their diet and nutritional intake. Therefore, it is essential to accurately assess dietary intake. In conclusion, the 120-item FFQ showed good reproducibility and acceptable validity for most food and nutrient intakes. It can be used to measure dietary intake in children aged 6–12 in western China. More work is required to validate whether this FFQ is appropriate in other regions or age groups.

## Figures and Tables

**Table 1:** Demographic and anthropometric characteristics of 133 children.

Characteristics	Total	Male (*n* = 68)	Female (*n* = 65)	^a^*p*-Value
Age (years, mean ± SD)	9.3 ± 0.9	9.5 ± 0.7	9.1 ± 1.0	0.05
6–9 years	65 (48.9)	28 (41.2)	37 (56.9)	0.07
9–12 years	68 (51.1)	40 (58.8)	28 (43.1)	
Height (cm, mean ± SD)	139.3 ± 8.5	141.2. ± 9.2	137.3 ± 7.4	<0.01
Weight (kg, mean ± SD)	35.1 ± 9.4	37.7 ± 10.1	32.4 ± 7.8	<0.01
^b^ Body mass index (BMI) (kg/m^2^, mean ± SD)	18.0 ± 3.3	18.7 ± 3.7	17.2 ± 2.6	0.07
Underweight (*n*, %)	6 (4.5)	3 (4.4)	3 (4.6)	0.23
Normal weight (*n*, %)	83 (62.4)	37 (54.4)	46 (70.8)	
Overweight (*n*, %)	23 (17.3)	14 (20.6)	9 (13.8)	
Obese (*n*, %)	21 (15.8)	14 (20.6)	7 (10.8)	
Father’s education level (*n*, %)				
Junior high school or below	66 (49.6)	38 (55.9)	28 (43.0)	0.06
Senior high school	29 (21.8)	17 (25.0)	12 (18.5)	
University degree or above	38 (28.6)	13 (19.1)	25 (38.5)	
Mother’s education level (*n*, %)				
Junior high school or below	58 (43.6)	33 (48.5)	25 (38.5)	0.32
Senior high school	32 (24.1)	17 (25.0)	15 (23.0)	
University degree or above	43 (32.3)	18 (26.5)	25 (38.5)	
^c^ Average family income (*n*, %)				
≤5000	11 (8.3)	7 (10.3)	4 (6.2)	0.27
5001–10,000	77 (57.9)	42 (61.8)	35 (53.8)	
>10,000	45 (33.8)	19 (27.9)	26 (40.0)	

^a^*p*-values from Student’s *t*-test or Mann–Whitney U test (continuous variables), and from Chi-square (categorical variables). ^b^ BMI cut-off points were based on Chinese standard for children and adolescents. ^c^ CNY/month, CNY 1 = USD 0.15.

**Table 2:** Reproducibility study: Median daily food intake, correlation coefficients and the agreement between FFQ1and FFQ2 in 133 children.

Food Groups (g/day)	Median (P25, P75)		^a^*p*-Value	Correlation Coefficient		Agreement (%)			^d^ K
	FFQ1	FFQ2		^b^ r_s_	^c^ r_i_	Same Quartile	Same or Adjacent Quartile	Extreme Quartile	
Cereals and potatoes	243 (193, 276)	218 (182, 279)	0.03	0.69 **	0.71 **	52.6	83.4	2.3	0.62
Meat	72 (51, 107)	76 (49, 113)	0.50	0.67 **	0.72 **	56.4	71.7	1.5	0.67
Aquatic products	7 (2, 12)	7 (3, 13)	0.71	0.52 **	0.40 **	46.6	83.4	5.3	0.49
Eggs	26 (11, 42)	26 (11, 42)	0.40	0.77 **	0.40 **	72.2	91.7	1.5	0.75
Milk and milk products	123 (77, 201)	121 (68, 204)	0.53	0.84 **	0.85 **	69.9	94.0	0.8	0.80
Soy and soy products	9 (6, 12)	8 (4, 11)	0.02	0.60 **	0.55 **	50.4	85.0	3.0	0.57
Vegetables	214 (140, 334)	230 (143, 337)	0.83	0.30 **	0.44 **	34.6	74.4	8.3	0.27
Fruits	164 (109, 286)	204 (112, 356)	<0.01	0.63 **	0.50 **	48.9	84.2	0.8	0.59
Nuts	4 (2, 10)	4 (1, 12)	0.47	0.45 **	0.45 **	40.6	77.4	6.0	0.38
Snacks	8 (4, 16)	11 (6, 19)	0.03	0.56 **	0.48 **	51.1	84.2	3.0	0.56
Beverages	52 (34, 122)	78 (34, 154)	0.02	0.38 **	0.20 *	36.1	72.9	7.5	0.35
Oil	17 (11, 21)	14 (10, 21)	0.17	0.52 **	0.54 **	56.4	73.7	0.0	0.49

^a^*p*-Value: Wilcoxon signed rank test. ^b^ r_s_: Spearman correlation coefficients. ^c^ r_i_: Intra-class correlation coefficients. ^d^ K: weighted kappa. * *p* < 0.05, ** *p* < 0.01.

**Table 3:** Reproducibility study: Median daily food intake, correlation coefficients and the agreement between FFQ1and FFQ2 in 133 children.

Energy and Nutrients	Median (P25, P75)		^a^*p*-Value	Correlation Coefficient		Agreement (%)			^d^ K
	FFQ1	FFQ2		^b^ r_s_	^c^ r_i_	Same Quartile	Same or Adjacent Quartile	Extreme Quartile	
Energy (kcal/day)	1623.3 (1400.2, 1877.8)	1610.1 (1319.6, 1952.5)	0.83	0.65 **	0.63 **	52.6	88.7	1.5	0.65
Protein (g/day)	57.2 (47.4, 67.8)	56.8 (45.5, 68.9)	0.97	0.66 **	0.68 **	48.9	89.5	1.5	0.64
Fat (g/day)	59.0 (51.3, 70.1)	58.2 (47.0, 72.0)	0.20	0.60 **	0.72 **	41.3	84.2	2.3	0.53
Carbohydrate (g/day)	223.7 (182.1, 267.4)	223.0 (177.8, 276.7)	0.73	0.65 **	0.58 **	53.4	89.5	2.3	0.62
Dietary fiber (g/day)	12.1 (9.2, 16.2)	12.4 (8.3, 17.0)	0.86	0.52 **	0.52 **	40.6	78.9	2.3	0.47
Cholesterol(mg/day)	275.5 (200.2, 408.8)	276.5 (184.1, 413.2)	0.61	0.82 **	0.52 **	60.1	94.0	0.8	0.73
Vitamin A (μgRAE/day)	401.9 (294.3, 521.7)	360.8 (283.5, 520.4)	0.44	0.47 **	0.37 **	36.8	82.7	4.5	0.46
Vitamin E (mg/day)	19.2 (15.4, 23.5)	19.2 (17.8, 23.4)	0.63	0.58 **	0.61 **	45.9	81.9	2.3	0.52
Thiamin (mg/day)	0.8 (0.7, 1.0)	0.8 (0.7, 1.0)	0.33	0.55 **	0.61 **	43.6	81.9	2.3	0.53
Riboflavin (mg/day)	0.9 (0.7, 1.1)	0.9 (0.7, 1.1)	0.67	0.57 **	0.52 **	48.1	83.4	2.3	0.56
Vitamin B6 (mg/day)	0.7 (0.5, 0.9)	0.7 (0.5, 1.0)	0.76	0.53 **	0.51 **	42.9	80.4	3.8	0.48
Vitamin B12 (μg/day)	1.5 (1.1, 2.0)	1.5 (1.1, 2.0)	0.30	0.68 **	0.61 **	53.4	89.5	2.3	0.66
Vitamin C (mg/day)	97.8 (59.4, 138.5)	94.9 (58.4, 162.0)	0.53	0.50 **	0.51 **	39.1	78.9	2.3	0.46
Pantothenic acid(mg/day)	2.0 (1.5, 2.9)	2.2 (1.4, 3.3)	0.35	0.46 **	0.42 **	42.9	79.7	3.8	0.47
Folate (μg/day)	215.8 (151.7, 275.6)	194.3 (147.6, 280.9)	0.30	0.53 **	0.49 **	39.8	80.5	3.0	0.47
Niacin (mg/day)	12.7 (10.6, 15.8)	12.8 (10.5, 17.0)	0.74	0.59 **	0.66 **	44.4	84.2	2.3	0.55
Calcium (mg/day)	441.1 (362.6, 583.0)	443.8 (336.9, 581.5)	0.80	0.60 **	0.49 **	51.9	85.0	1.5	0.60
Phosphorus (mg/day)	932.8 (784.4, 1103.9)	933.3 (775.5, 1139.3)	0.90	0.64 **	0.65 **	42.1	89.5	2.3	0.60
Potassium (mg/day)	1891.9 (1454.5, 2258.6)	1901.7 (1421.2, 2469.6)	0.70	0.56 **	0.57 **	45.1	83.5	2.3	0.54
Sodium (mg/day)	874.9 (659.8, 1119.3)	870.6 (611.7, 1072.7)	0.09	0.59 **	0.61 **	45.9	84.2	2.3	0.55
Magnesium (mg/day)	263.5 (214.4, 324.4)	259.2 (207.8, 338.4)	0.96	0.56 **	0.56 **	47.4	81.9	3.0	0.52
Iron (mg/day)	14.1 (11.5, 17.0)	13.6 (10.8, 17.5)	0.64	0.62 **	0.60 **	44.4	85.7	1.5	0.58
Iodine (μg/day)	71.0 (54.2, 94.1)	62.4 (47.5, 93.4)	<0.01	0.60 **	0.54 **	41.3	87.2	2.3	0.57
Zinc (mg/day)	7.8 (6.5, 9.4)	7.9 (6.3, 9.7)	0.85	0.60 **	0.64 **	44.4	81.2	2.3	0.52
Selenium (μg/day)	29.3 (24.0, 35.7)	30.0 (22.6, 36.6)	0.37	0.72 **	0.75 **	53.4	89.5	1.5	0.66
Copper (mg/day)	1.2 (0.9, 1.6)	1.2 (0.9, 1.7)	0.39	0.60 **	0.59 **	43.6	82.7	1.5	0.54
Manganese (mg/day)	3.8 (3.2, 4.7)	3.8 (3.1, 4.7)	0.78	0.64 **	0.66 **	45.9	84.2	2.3	0.56

^a^*p*-Value: Wilcoxon signed rank test. ^b^ r_s_: Spearman correlation coefficients. ^c^ r_i_: Intra-class correlation coefficients. ^d^ K: Weighted kappa. ** *p* < 0.01.

**Table 4:** Relative validity study: Median daily food intake, correlation coefficients, agreement and weighted kappa between FFQ1 and the average of three 24 h recalls in 133 children.

Food Groups (g/day)	Median (P25, P75)		^a^*p*- Value	Correlation Coefficient		Agreement (%)			^d^ K
	FFQ1	24HR		^b^ r_s_	^c^ r_e-adj_	Same Quartile	Same or Adjacent Quartile	Extreme Quartile	
Cereals and potatoes	243 (193, 276)	262 (212, 304)	<0.01	0.35 **	0.21 *	30.8	72.2	5.3	0.29
Meat	72 (51, 107)	62 (38, 94)	<0.01	0.24 **	0.24 **	28.6	69.2	6.0	0.23
Aquatic products	7 (2, 12)	0.5 (0, 1.4)	<0.01	0.15	0.15	37.6	75.2	9.8	0.12
Eggs	26 (11, 42)	31 (17, 49)	0.02	0.17 *	0.12	30.8	67.7	11.3	0.14
Milk and milk products	123 (77, 201)	76 (0, 133)	<0.01	0.37 **	0.37 **	36.8	77.4	4.5	0.37
Soy and soy products	9 (6, 12)	14 (7.6, 20)	<0.01	0.36 **	0.30 **	33.1	77.4	5.3	0.33
Vegetables	214 (140, 334)	184 (137, 235)	<0.01	0.16	0.13	28.6	77.4	7.5	0.26
Fruits	164 (109, 286)	81 (43, 132)	<0.01	0.32 **	0.26 **	34.6	71.4	8.3	0.24
Nuts	4 (2, 10)	3 (0, 6)	<0.01	0.05	0.07	26.3	65.4	9.8	0.05
Snacks	8 (4, 16)	5 (0, 21)	0.09	0.15	0.15	30.1	64.7	12.3	0.14
Beverages	52 (34, 122)	25 (0, 67)	<0.01	0.02	0.02	37.0	75.5	8.2	0.06
Oil	17 (11, 21)	11 (9, 13)	<0.01	0.11	0.13	30.9	70.7	7.5	0.13

^a^*p*-Value: Wilcoxon signed rank test. ^b^ r_s_: Spearman correlation coefficients. ^c^ r_e-adj_: Energy-adjusted correlation coefficients. ^d^ K: Weighted kappa. * *p* < 0.05, ** *p* < 0.01.

**Table 5:** Relative validity study: Median daily energy and nutrient intake, correlation coefficients between FFQ1 and the average of three 24 h recalls in 133 children.

Energy and Nutrients	Median (P25, P75)		^a^*p*-Value	Correlation Coefficient		
	FFQ1	24HR		^b^ r_s_	^c^ r_e-adj_	^d^ r _de-att_
Energy (kcal/day)	1623.3 (1400.2, 1877.8)	1425.0 (1235.0, 1604.0)	<0.01	0.44 **	-	0.48 **
Protein (g/day)	57.2 (47.4, 67.8)	50.6 (44.4, 57.0)	<0.01	0.37 **	0.30 **	0.42 **
Fat (g/day)	59.0 (51.3, 70.1)	44.0 (34.4, 52.1)	<0.01	0.31 **	0.28 **	0.36 **
Carbohydrate (g/day)	223.7 (182.1, 267.4)	204.1 (177.7, 240.6)	<0.01	0.45 **	0.36 **	0.48 **
Dietary fiber (g/day)	12.1 (9.2, 16.2)	8.3 (7.0, 9.7)	<0.01	0.35 **	0.28 **	0.39 **
Cholesterol(mg/day)	275.5 (200.2, 408.8)	243.0 (194.0, 356.5)	0.24	0.22 *	0.18 *	0.26 **
Vitamin A (μgRAE/day)	401.9 (294.3, 521.7)	219.0 (165.0, 273.5)	<0.01	0.08	0.02	0.09
Vitamin E (mg/day)	19.2 (15.4, 23.5)	16.7 (14.2, 16.6)	<0.01	0.21 *	0.22 *	0.25 **
Thiamin (mg/day)	0.8 (0.7, 1.0)	0.8 (0.6, 0.9)	0.001	0.25 **	0.22 *	0.29 **
Riboflavin (mg/day)	0.9 (0.7, 1.1)	0.7 (0.5, 0.8)	<0.01	0.33 **	0.30 **	0.38 **
Vitamin C (mg/day)	97.8 (59.4, 138.5)	55.7 (41.1, 76.9)	<0.01	0.21 *	0.15	0.25 **
Folate (μg/day)	215.8 (151.7, 275.6)	84.2 (68.4, 106.7)	<0.01	0.33 **	0.31 **	0.38 **
Niacin (mg/day)	12.7 (10.6, 15.8)	11.3 (9.7, 12.9)	<0.01	0.26 **	0.20 *	0.30 **
Calcium (mg/day)	441.1 (362.6, 583.0)	328.0 (277.9, 428.5)	<0.01	0.39 **	0.32 **	0.44 **
Phosphorus (mg/day)	932.8 (784.4, 1103.9)	754.0 (672.0, 856.5)	<0.01	0.35 **	0.33 **	0.40 **
Potassium (mg/day)	1891.9 (1454.5, 2258.6)	1334.0 (1172.0, 1509.5)	<0.01	0.21 *	0.12	0.24 **
Sodium (mg/day)	874.9 (659.8, 1119.3)	491.4 (370.5, 654.0)	<0.01	0.28 **	0.17 *	0.33 **
Magnesium (mg/day)	263.5 (214.4, 324.4)	235.0 (212.0, 264.5)	<0.01	0.32 **	0.28 **	0.36 **
Iron (mg/day)	14.1 (11.5, 17.0)	13.7 (12.1, 15.4)	0.80	0.29 **	0.22 *	0.32 **
Iodine (μg/day)	71.0 (54.2, 94.1)	35.6 (25.8, 52.6)	<0.01	0.20 *	0.07	0.23 **
Zinc (mg/day)	7.8 (6.5, 9.4)	7.2 (6.4, 7.9)	<0.01	0.29 **	0.26 **	0.33 **
Selenium (μg/day)	29.3 (24.0, 35.7)	27.7 (23.7, 32.8)	0.16	0.31 **	0.18 *	0.36 **
Copper (mg/day)	1.2 (0.9, 1.6)	1.0 (0.9, 1.2)	<0.01	0.19 *	0.10	0.22 *
Manganese (mg/day)	3.8 (3.2, 4.7)	3.6 (3.1, 4.2)	0.001	0.44 **	0.35 **	0.47 **

^a^*p*-Value: Wilcoxon signed rank test. ^b^ r_s_: Spearman correlation coefficients. ^c^ r_e-adj_: Energy-adjusted correlation coefficients. ^d^ r _de-att_: De-attenuated and un-energy adjusted correlation coefficients. * *p* < 0.05, ** *p* < 0.01.

**Table 6:** Relative validity study: Agreement and weighted kappa for daily energy and nutrient intakes between FFQ1 and the average of three 24 h recalls in 133 children.

Energy and Nutrients	Agreement by Quartile (%)			^a^ K	^b^ Agreement by LoA (%)
	Same Quartile	Same or Adjacent Quartile	Extreme Quartile		
Energy	38.3	75.9	4.5	0.38	95.5
Protein	31.6	69.9	3.8	0.35	95.5
Fat	35.3	75.2	6.8	0.31	95.5
Carbohydrate	39.1	77.4	5.3	0.38	94.7
Dietary fiber	33.8	75.2	8.3	0.28	94.0
Cholesterol	23.3	74.4	9.0	0.21	94.7
Vitamin A	28.6	63.2	9.8	0.08	93.2
Vitamin E	25.6	66.9	8.3	0.15	96.2
Thiamin	30.8	71.4	9.0	0.23	94.7
Riboflavin	30.1	74.4	6.0	0.31	94.7
Vitamin C	29.3	66.9	7.5	0.18	95.5
Folate	27.8	76.7	5.3	0.33	97.0
Niacin	32.3	69.2	5.3	0.26	94.7
Calcium	39.8	75.9	4.4	0.39	94.0
Phosphorus	34.6	72.9	3.0	0.36	95.5
Potassium	26.3	70.7	9.8	0.16	94.7
Sodium	31.6	72.9	6.8	0.27	94.0
Magnesium	30.8	70.7	6.0	0.26	97.0
Iron	30.8	72.2	6.8	0.26	94.7
Iodine	30.1	66.2	10.5	0.11	98.5
Zinc	34.6	72.9	9.0	0.26	96.2
Selenium	36.8	73.7	6.8	0.30	94.7
Copper	30.8	63.9	10.5	0.10	96.2
Manganese	38.3	78.9	5.3	0.41	96.2

^a^ K: Weighted kappa. ^b^ Agreement by LoA: Overall proportion of agreement limits between both questionnaires. Corresponding to Bland–Altman plots.

## Supplementary Materials

The following supporting information can be downloaded at: https://www.mdpi.com/article/10.3390/nu15040856/s1, Table S1. Foods and drinks were used in the study; Table S2. Sensitivity analysis: Correlation coefficients, agreement and weighted kappa for daily energy and nutrient intake from two FFQs, 24-h recall in males and females, respectively.
