# Supplementary material for: Reproducibility and Validity of a Semi-Quantitative Food Frequency Questionnaire for Children Aged 6–12 in Western China
Source: Nutrients. 2023 Feb 8;15(4):856. doi: 10.3390/nu15040856 (PMC9959506; doi:10.3390/nu15040856)
Supplement: Supplementary file 1 [file nutrients-15-00856-s001.zip › nutrients-2143346-supplementary.pdf]

Table S1. Foods and drinks were used in the study

Food groups (12)	Food items (120)
Cereals and potatoes	rice, noodles, steamed buns, pie, cold noodles, rice noodles, Chinese hamburger, steamed stuffed bun, dumpling, wonton, sweet dumpling, rice porridge, millet congee, oatmeal, vermicelli, corn, potato, sweet potato, purple sweet potato, bread
Meat	pork, pork rib, beef, mutton, chicken breast, drumstick, chicken wing, duck, blood, liver, sausage, lunchmeat, ham
Eggs	chicken eggs
Aquatic products	grass carp, hairtail, dried small shrimp, shrimp
Milk and milk products	fresh milk, yogurt, cheese
Soy and soy products	tofu, dried tofu, soybean milk
Vegetables	konjac, turnip, carrot, cucumber, wax gourd, summer squash, pumpkin, Chinese yam, lotus root, tomato, pepper, green bean, bean sprouts, cabbage, cauliflower, broccoli, pakchoin, spinach, water spinach, leaf lettuce, Chinese cabbage, romaine lettuce, lettuce, celery, leek, chrysanthemum, eggplant, onion, shitake, mushroom, flammulina velutipes, oyster mushroom, pleurotus eryngii, kelp, laver, fungus
Fruits	apple, banana, pear, orange, strawberry, pineapple, cherry, muskmelon, watermelon, cantaloupe, peach, grape, blueberry, pitaya, mango, durian, mangosteen, pomegranate, kiwi fruit, pomelo, jujube
Nuts	walnut, sunflower seed, pine nut, cashew, peanut
Snacks	chocolate, candy, biscuit, cake, ice cream, preserved fruit, puffed food
Beverages	cola, juice, tea, milk drinks, sports drinks, bubble tea
Oil	canola oil

Table S2. Sensitivity analysis: Correlation coefficients, agreement and weighted kappa for daily energy and nutrient intake from two FFQs, 24-h recall in males and females, respectively.

**Energy and nutrients**	**FFQ1 vs. FFQ2**						**FFQ1 vs. 24HR**					
	**Male(*n*=68)**			**Female(*n*=65)**			**Male(*n*=68)**			**Female(*n*=65)**		
	[a]r_s	[b]**Agreement by quartile (%)**	[c]**K**	r_s	**Agreement by quartile (%)**	**K**	r_s	**Agreement by quartile (%)**	**K**	r_s	**Agreement by quartile (%)**	**K**
Energy	0.71**	88.2	0.68	0.57**	89.2	0.58	0.42**	79.4	0.37	0.42**	73.8	0.42
Protein	0.71**	89.7	0.64	0.62**	89.2	0.61	0.39**	76.5	0.35	0.31**	70.8	0.28
Fat	0.70**	85.3	0.59	0.48**	80.0	0.44	0.41**	79.4	0.36	0.20	64.6	0.13
Carbohydrate	0.68**	89.7	0.68	0.62**	87.7	0.57	0.31**	73.5	0.24	0.61**	86.2	0.53
Dietary fiber	0.59**	82.3	0.58	0.45**	80.0	0.43	0.29*	75.0	0.29	0.39**	76.9	0.36
Cholesterol	0.85**	97.1	0.78	0.80**	92.3	0.77	0.15	70.6	0.18	0.28*	76.9	0.30
Vitamin A	0.42**	79.4	0.40	0.52**	83.1	0.48	0.04	61.8	0.02	0.12	64.6	0.15
Vitamin E	0.63**	83.8	0.59	0.51**	76.9	0.45	0.16	63.2	0.09	0.21	67.7	0.13
Thiamin	0.57**	83.8	0.55	0.52**	77.9	0.52	0.24	73.5	0.23	0.25*	69.2	0.19
Riboflavin	0.63**	86.8	0.60	0.51**	81.5	0.51	0.32**	75.0	0.27	0.32**	73.8	0.33
Vitamin C	0.51**	79.4	0.46	0.48**	73.8	0.40	0.18	66.2	0.17	0.24	69.2	0.22
Folate	0.57**	80.9	0.49	0.47**	78.5	0.44	0.41**	82.4	0.44	0.23	72.3	0.25
Niacin	0.62**	85.3	0.55	0.56**	83.1	0.55	0.34**	76.5	0.38	0.17	61.5	0.14
Calcium	0.62**	82.3	0.55	0.58**	87.7	0.61	0.41**	79.4	0.38	0.37**	72.3	0.32
Phosphorus	0.69**	91.2	0.68	0.59**	89.2	0.56	0.41**	76.5	0.39	0.28*	67.7	0.31
Potassium	0.61**	85.3	0.59	0.51**	84.6	0.53	0.17	70.6	0.18	0.22	70.8	0.20
Sodium	0.67**	86.7	0.61	0.54**	80.0	0.49	0.29*	70.6	0.22	0.27*	70.8	0.25
Magnesium	0.55**	76.5	0.49	0.55**	86.1	0.55	0.31**	80.9	0.33	0.37**	73.8	0.37
Iron	0.69**	88.2	0.64	0.57**	83.1	0.56	0.44**	82.4	0.42	0.16	67.7	0.08
Iodine	0.56**	85.3	0.49	0.65**	86.1	0.63	0.21	70.6	0.17	0.15	64.6	0.16

Zinc	0.64**	86.8	0.61	0.56**	80.0	0.47	0.41**	77.9	0.27	0.16	66.2	0.12
Selenium	0.75**	91.2	0.67	0.70**	89.2	0.64	0.26*	69.1	0.23	0.29*	73.8	0.36
Copper	0.62**	80.9	0.55	0.58**	83.1	0.54	0.25*	67.6	0.08	0.17	66.2	0.07
Manganese	0.67**	86.8	0.66	0.60**	87.7	0.55	0.42**	75.0	0.24	0.41**	78.5	0.40

[a]r_s: Spearman correlation coefficients

[b]Agreement by quartile: The percentage of the subjects classified in the same or adjacent quartile

[c]K: Weighted kappa

*$p < 0.05$, **$p < 0.01$
